# Mechanisms of blood flow restriction training for knee pain: a mini review

**DOI:** 10.3389/fphys.2025.1542322

**Published:** 2025-02-13

**Authors:** Shi-Yu Xie, Xue Jiang, Jia-Bin Yuan, Jing Luo, Shun Song, Hao-Yu Hu

**Affiliations:** ^1^ Department of Sport Rehabilitation, Shanghai University of Sport, Shanghai, China; ^2^ Department of Orthopedics, Changhai Hospital, Naval Medical University, Shanghai, China; ^3^ Department of Rehabilitation Medicine, Xi’an Physical Education University, Xian, China; ^4^ Department of Physical education Shanghai Jiao Tong University, Shanghai, China; ^5^ Department of Rehabilitation Medicine, Shanghai Shangti Orthopaedic Hospital, Shanghai, China

**Keywords:** BFRT, arthralgia, patellar, injuries, inflammation

## Abstract

Knee pain, affecting an estimated 654 million people worldwide, so blood flow restriction training (BFRT) is catching the spotlight as an effective intervention. Evidence continues to demonstrate the effectiveness of BFRT in managing knee pain. However, the mechanism by which BFRT alleviates knee pain remains unclear, thereby limiting its application in clinical pain management. This study aims to elucidate the underlying mechanisms of BFRT to better understand its efficacy in treating knee pain. This review will discuss the influence of muscle hypertrophy, endogenous opioid system, endocannabinoids, inflammation regulation, and conditional pain regulation on BFRT treatment of knee pain. Current studies on BFRT have limitations, such as small sample sizes, relatively low-quality evidence, and lack of mechanistic studies. Therefore, further research on BFRT is needed, particularly high-quality and large-sized randomized controlled trials.

## 1 Introduction

Knee pain affects millions worldwide, diminishing quality of life and mobility. As conventional treatments demonstrate limitations, blood flow restriction training (BFRT) emerges as a promising alternative. Approximately 5% of all primary care visits in adults are related to knee pain, which reduces function and mobility (Duong, et al., 2023). The prevalence of knee pain and symptomatic knee osteoarthritis (KOA) has increased over the course of 20 years, approximately doubling in women and tripling in men, and they now account for almost 4 million primary care visits per year (Nguyen et al., 2011). Patellar Tendinopathy (PT), anterior cruciate ligament (ACL) injury, and KOA are some of the factors that contribute to knee pain. The most typical reason for knee pain in persons 45 years and older is KOA (Duong, et al., 2023). More than half of the elderly (Kim et al., 2011) and 19%–31% of adolescents (Smith et al., 2018) currently have knee pain, and long-term knee pain may lead to a decline in quality of life (Crossley et al., 2016; Deshpande et al., 2016).

For various diseases that cause knee pain, exercise is recommended for pain management (Kolasinski et al., 2020; Uthman et al., 2013). Different types of exercise have been shown to have a positive effect on chronic pain (Fransen et al., 2015; Goh et al., 2019; Hussain et al., 2016; Imoto et al., 2019; Kan et al., 2019; van den Ende et al., 2000). Several authoritative organizations, including the American Academy of Orthopedic Surgeons (Jevsevar, 2013), the Osteoarthritis Research Society International (Bannuru et al., 2019), and the American College of Rheumatology (Hochberg et al., 2012), have noted that the enhancement of lower limb muscle strength could effectively reduce pain. BFRT was defined as a method of blood flow restriction combined with resistance training (Barber-Westin and Noyes, 2019). The benefit of BFRT is that loads around 30% of maximum force provide outcomes that are comparable to or greater than 80% of maximum force (Jack et al., 2022; Minniti, et al., 2020). BFRT has been demonstrated to be a secure and efficient training technique that builds muscular growth and strength (Loenneke et al., 2012b; Minniti et al., 2020).

To date, several reviews and meta-analyses have investigated the clinical efficacy of BFRT in the treatment of knee disease. A systematic review showed evidence of a potential benefit of perioperative BFRT for muscular mass in those undergoing ACL reconstruction (Lu et al., 2020). High-load BFRT could considerably enhance muscular strength in individuals with knee injuries according to the meta-analysis of Li et al., and low-load BFRT could remarkably reduce pain intensity (Li et al., 2021). Recent literature has demonstrated the efficacy of BFRT in alleviating pain associated with PT (Burton and McCormack, 2022). Besides, a systematic review of KOA showed that BFRT was effective in increasing muscle strength and reducing pain (Pitsillides et al., 2021). For both patients with KOA and those with ACL injuries, BFRT can provide more effective pain relief. Although the current review supports the role of BFRT, less clinical evidence exists and more studies are needed to draw more definitive conclusions. However, all of these reviews only described the role of BFRT and not the underlying mechanisms that BFRT is used for knee pain. To the authors’ knowledge, only one review has mentioned the mechanism of pain reduction after BFRT (Song et al., 2021), and the study mentioned that pain reduction after BFRT may be related to activation of endogenous opioid and cannabinoid system, high threshold motor unit recruitment, cardiovascular system, and conditioned pain modulation. However, no studies have reviewed the underlying mechanisms of BFRT in the treatment of knee pain. This review explores the underlying mechanisms of BFRT to provide a comprehensive understanding of its role in knee pain management, thereby providing a theoretical foundation for future research on the impact of BFRT on patients with knee pain.

## 2 Effects of blood flow restriction training on knee pain

BFRT have been increasingly used to treat knee pain. Mahmoud et al. recruited 35 subjects and observed that a combination of 70% of total occlusion pressure with 30% 1-RM training was effective in relieving pain (Mahmoud et al., 2021). Giles et al. recruited 79 people to perform BFRT or resistance training three times a week for 8 weeks. The results showed that the BFRT group experienced a 93% reduction in daily life pain over 8 weeks, but the worst pain was not statistically different between the two groups (Giles et al., 2017). Knee pain was also lower with BFRT during and at 24 h post-training after 8 weeks of twice-weekly BFRT than with high-load resistance training (HL-RT) (Hughes et al., 2019). Another study compared the clinical effects of 3-week conventional resistance training and twice-daily BFRT. The results showed that the pain scores during training were considerably lower over time in the BFRT group (Ladlow et al., 2018). Ferraz et al. also supported that BFRT could relieve knee pain and reduce joint stress compared with HL-RT (Ferraz et al., 2018). In addition, Tennent et al. recruited 17 people and assigned them to either the BFRT group or the standard physical therapy group for 12 sessions. The Knee Injury and Osteoarthritis Outcome Scores improved obviously on several subscales in both groups, with the BFRT group showing 1.5–2 times improvement in all subscales (Tennent et al., 2017). However, research by Segal et al. showed that low-load BFRT (LL-BFRT) did not considerably improve knee pain (Segal et al., 2015). In this study, quadriceps strength exhibited a significant increase; however, no substantial improvements were observed in quadriceps volume or pain levels. This may potentially be attributed to insufficient training frequency or intensity (Segal et al., 2015). Despite the varying results observed, integrating these findings with an exploration of the underlying mechanisms will lead to more robust and substantiated conclusions. Table 1 summarizes the study characteristics of BFRT for knee pain. BFRT was effective in reducing knee pain in the majority of cases.

**TABLE 1:** Major characteristics of studies focused on blood flow restriction training for knee pain.

Reference	Sample size	Age	Intervention	Duration of trial period	Pain outcomes	Outcome assessment	Result	Change
Mahmoud et al. (2021)	35subjects, 2G	G1: 59.05 ± 1.83 G2: 60.17 ± 2.09	G1: partial BFRT (50%) G2: partial BFRT (70%)	Treatment intervention continued for 8 weeks	VAS	Pain was evaluated before and after the treatment intervention	70% combination with 30% 1RM could be beneficial for improving pain	↑
Giles et al. (2017)	G1: n = 40 G2: n = 39	G1: 28.5 ± 5.2 G2: 26.7 ± 5.5	G1: LL-BFRT G2: LL-RT	Performed the exercises three times per week over 8 weeks	VAS	Worst pain in the past week and pain with activities of daily living	here was a 93% greater reduction in pain with ADL over 8 weeks in the BFRT group	↑
Hughes et al. (2019)	G1: n = 14 G2: n = 14	G1: 29 ± 7 G2: 29 ± 7	G1: BFRT G2: HL-RT	8 weeks of twice weekly training	Borg’s CR-10 Pain scale	Assess pain during training and 24 h post-training	Knee pain was lower with BFR-RT during and at 24 h post-training with BFR-RT for all sessions	↑
Ladlow et al. (2018)	G1: n = 14 G2: n = 14	G1: 33 ± 6 G2: 28 ± 7	G1: conventional resistance training G2: LL-BFRT	3-week rehabilitation program	VAS	During the exercise and 5 min post-exercise	The Pain scores during training reduced significantly over-time in the LL-BFR group	↑
Ferraz et al. (2018)	G1: n = 16 G2: n = 16 G3: n = 16	G1: 59.9 ± 4 G2: 60.7 ± 4 G3: 60.3 ± 3	G1: LL- BFRT G2: LL-RT G3: HL-RT	Exercise occurred two times a week for 12 weeks	WOMAC	Before and after the protocol	BFRT was also able to improve pain while inducing less joint stress	↑
Tennent et al. (2017)	G1: n = 10 G2: n = 7	G1: 37.0 (30–46.2) G2: 37.0 (32–47)	G1: BFRT G2:standard physical therapy	12 sessions of therapy	KOOS	Before and after the protocol	BFRT group improved more significantly than control group across all subscales	↑
Segal et al. (2015)	G1: n = 19 G2: n = 21	G1: 56.1 ± 5.9 G2: 54.6 ± 6.9	G1: LL-RT G2: LL- BFRT	Exercise occurred three times a week for 4 weeks	KOOS	Pain was evaluated before and after the participation	Knee related pain did not significantly differ between groups	NC

G, group; BFRT, blood flow restriction training; HL, heavy load; LL, low load; RT, resistance training; VAS, visual analogue scale; WOMAC, The Western Ontario and McMaster Universities; KOOS, knee injury and osteoarthritis outcome score; ↑ significant increase, NC, no change.

## 3 Mechanism of high-load resistance training mechanism for treating knee pain

### 3.1 Muscle hypertrophy

A strong quadriceps muscle is generally believed to provide structural stability and assistance to damaged and degenerated knees and thus effectively relieve knee pain (Lee et al., 2018). Meanwhile, muscle atrophy is also a serious complication of various knee joint diseases (Bannuru et al., 2019; Hochberg et al., 2012; Jevsevar, 2013). BFRT could induce muscle hypertrophic adaptation and increase muscle strength at the same time (Patterson et al., 2019). At present, muscle hypertrophy is considered to be mainly caused by mechanical tension and metabolic stress, and a large number of studies have shown that mechanical tension is the main mechanism of muscle growth. However, most BFRTs only use low-load resistance (30%–50% 1-RM), Therefore, BFRT generally could not induce mechanisms related to mechanical tension (Pearson and Hussain, 2015). Goto et al. (2005) conducted two groups of exercise programs (3–5 groups of 10 repetitions; the intensity was only 10-RM; the rest time between groups was 1 min; and the movements used pull-ups, shoulder presses, and bilateral knee extensions) for a completely consistent experimental comparison. The only difference between the acute and chronic effects was that one of the sets included a 30-s break at the midpoint of each set to alter exercise-induced metabolic stress. Acute hormonal responses were measured for both regimens followed by 12 weeks of resistance training. The results showed that the no-rest regimen induced stronger blood lactate concentration, growth hormone, epinephrine, and norepinephrine responses than the rest regimen. After 12 weeks of training, the no-rest program group had considerably increased maximal strength, maximal isometric strength, muscular endurance, and muscle cross-sectional area (CSA) in knee extension compared with the rest program group, revealing a link between metabolic stress and muscle hypertrophy.

Numerous studies have also shown that metabolic stress linked to BFRT may have hypertrophic effects, in which low-load resistance training (LL-RT, 30%–50% 1-RM) resulted in a remarkable increase in muscle CSA. In addition, literature has reported a direct relationship between other indicators of exercise metabolic stress and muscle hypertrophy after LL-BFRT (20% 1-RM). This finding may highlight a major role for metabolic stress causing muscle hypertrophic adaptations after BFRT. Exercise-induced metabolic stress has been hypothesized to mediate muscle hypertrophy through various mechanisms, including increased systemic hormone production, increased recruitment of fast-twitch fibers, cellular swelling, muscle damage, and increased reactive oxygen species (ROS) production. All of these factors are hypothesized to mediate muscle protein signaling and/or satellite cell proliferation to induce muscle growth.

This level of metabolic stress is also amplified under BFRT conditions. Blood lactate concentrations (Kon et al., 2012; Takarada et al., 2000) and Growth hormone increases were stronger and greater following LL-RT than after the same workout program without BFRT (Takarada et al., 2000). A number of studies have demonstrated the potential hypertrophic effects of metabolic stress associated with BFRT, in which LL-RT (30%–50% 1-RM) resulted in a evident increase in muscle CSA (Takarada et al., 2000; Takarada et al., 2002; Takarada et al., 2004). In addition, the literature has reported a direct relationship between other indicators of exercise metabolic stress and muscle hypertrophy after LL-BFRT (20% 1RM) (Takada et al., 2012). This may highlight a major role for metabolic stress causing muscle hypertrophic adaptations after BFRT. Exercise-induced metabolic stress is thought to mediate muscle hypertrophy via a variety of mechanisms, including increased systemic hormone production (Reeves et al., 2006), increased recruitment of fast-twitch fibers (Takarada et al., 2002), cellular swelling (Berneis, et al., 1999; Loenneke, et al., 2012a), muscle damage (Schoenfeld, 2013), and increased ROS production (Pope et al., 2013; Schiaffino et al., 2013), all of these are hypothesized to play a role in satellite cell proliferation and/or muscle protein signaling to promote muscular growth. It is also noteworthy that a recent review has highlighted the varying effects of BFRT on muscle hypertrophy across different training states. Highly trained individuals may achieve superior strength and hypertrophy gains through BFRT compared to their untrained counterparts (Geng, et al., 2024). This represents a significant advancement for individuals suffering from PT. PT is a degenerative condition that affects the patellar tendon, typically resulting from prolonged overstretching or repetitive overuse of the tendon (Burton and McCormack, 2022). This suggests that BFRT may yield superior outcomes for the treatment of PT.

### 3.2 Endogenous opioid system

Beta-endorphin (BE) is an opioid neuropeptide that is considered one of the important substances affecting exercise-induced hypoalgesia (EIH) (Rice et al., 2019; Vaegter and Jones, 2020). The circulating concentration of BE increases after human exercise, which results from the activation of the opioid system after stimulating afferent fibers in groups III and IV in contractile muscles (Thorén et al., 1990). This phenomenon triggers the release of BE by the pituitary gland and peripheral neurons functioning as an agonist for opioid receptors, which are widely distributed in the central and peripheral nervous systems’ descending pain control circuits. BEs provide analgesic effects by attaching to opioid receptors in presynaptic and postsynaptic nerve endings in the peripheral nervous system, thereby inhibiting the tachykinin substance P, which is known to play a major role in pain transmission. Considering that the central nervous system also contains opioid receptors, BEs could act as an analgesic through this system. Instead of blocking the tachykinin substance P in the central nervous system, BEs provide analgesia by boosting dopamine release (Sprouse-Blum et al., 2010). Studies have shown that taking opioid agonists, such as naloxone, may reduce hypoalgesia, affirming the part the opioid system plays in hypoalgesia (Haier et al., 1981). Hughes and Patterson (2020) compared the acute effects of LL-RT and BFRT on pain sensitivity. The pain threshold of the moving limb was found to be higher after low- and high-pressure BFRT and HL-RT than after LL-RT (26%–48% vs. 10%), and after BFRT40 (p < 0.01, d = 0.47) and BFRT80 (p > 0.01, d > 0.49) than after HL-RT, indicating that BFRT was involved in the opioid-mediated mechanisms in EIH. However, after 24 h of exercise, the BE levels of all four groups and the pressure pain thresholds (PPTs) after LLRE and HLRE returned to baseline levels. Moreover, the PPT of motor limbs after low- and high-pressure BFRT remained above baseline (15% and 24%, respectively). Therefore, EIH caused by BFRT may be associated with more than just endogenous opioid production.

### 3.3 Endocannabinoids

The endogenous cannabinoid (ECB) system belongs to the non-opioid mechanism (Koltyn, et al., 2014), which is situated in the central nervous system’s region responsible for processing pain (Hohmann and Suplita, 2006). Some studies suggest that the non-opioid mechanism may also be related to EIH (Vaegter et al., 2019). The ECB system is a neuromodulatory system that is composed of cannabinoid receptors and endogenous ligand agonists (including anandamide and 2-indoloylglycerol [2-AG]) found at pain-treating sites on the peripheral and spinal cord and in proteins that regulate their metabolism (Starowicz et al., 2013; Vaegter et al., 2019). Under stress conditions such as exercise, cells synthesize and rapidly release ECB, and ECB produces analgesic effects by binding to cannabinoid receptors (Feuerecker et al., 2012). Studies have shown elevated ECB concentrations following exercise, pointing to a potential role for the ECB system in EIH. This hypothesis are supports by the ECB receptors that activated during muscle contraction, which dense exist on Aδ and Cδ primary afferent fibers (Feuerecker et al., 2012). The receptor activated during muscle contraction, resulting in changes in circulating concentrations of ECB (Koltyn et al., 2014).

Current animal studies have shown that opioid or non-opioid hypoalgesia mechanisms could be elicited by adjusting the duration and intensity of exercise stressors (Starowicz et al., 2013). However, studies on BFRT have only measured the concentration of 2-AG and discovered that it remained constant over time (pre-exercise, 5 min post-workout, and 24 h post-workout) (Hughes and Patterson, 2020). Previous research has demonstrated that intense exercise does not alter circulating 2-AG concentrations, whereas another ECB (called anandamide) has been found to increase (Heyman, et al., 2012). Which could be based on their various metabolic pathways. As a result, how BFRT affects other ECB substances is unclear.

### 3.4 Inflammation

Under normal physiological conditions, the inflammatory response is the response of the immune system when harmful stimulus causes damage to the body (Weiss, 2008). In recent years, evidence showed that the inflammatory response has a remarkable effect on knee pain, such as increased inflammation, increased interleukin (IL)-1β, and increased macrophages in the subpatellar fat pad (Bastiaansen-Jenniskens et al., 2012), which could speed up the deterioration of the cartilage by allowing the deep-lying nerve fibers in the subchondral bone and cartilage to send pain signals to the brain (Adatia et al., 2012). Studies have revealed that elevated inflammatory molecules, such as IL-6, tumor necrosis factor-α (TNF-α), and MMP-13, were present in the knee synovial fluid of patients with KOA who are in pain (Runhaar et al., 2019). After treatment, the inflammatory molecules were remarkably reduced (Schell et al., 2017). Thus, synovial fluid inflammation is a major contributor to the emergence of KOA-related pain. These results suggested that reducing knee pain by suppressing inflammation may be a viable mechanism.

At present, a large number of animal experiments could prove that exercise training could reduce the expression of inflammatory molecules, such as IL-1β, caspase-3, and MMP-13, to effectively prevent inflammation (Adatia et al., 2012). However, the effect of BFRT on inflammatory biomarkers is minimal. BFRT only resulted in a 163% increase in the anti-inflammatory macrophage phenotype after 3 weeks as opposed to LL-RT (20% 1-RM) or HL-RT (70% 1-RM). In addition, the pro-inflammatory macrophage levels were higher in the LL-RT group than in the BFRT group 3 days after the end of the training period. An 18% decrease in plasma IL-6 before BFRT treatment to 180 min was also recorded, whereas no change was observed in the high-load control group (Nielsen et al., 2017). Biweekly BFRT after 8 weeks tended to lower the levels of inflammatory biomarkers after training (Kambič et al., 2019). Some evidence showed that BFRT may have anti-inflammatory properties. However, few research has been conducted in this area, and additional investigation is required to understand the precise mechanistic connection between BFRT and pro-inflammatory factors.

### 3.5 Triggering of conditional pain regulation (CPM)

CPM is a phenomenon known as “pain suppression” that is often used to assess the body’s ability to regulate pain (Kennedy et al., 2016). In human studies, CPM is usually triggered by the application of a nourishing harmful cold stimulus, which could lead to an acute ectopic decrease in pain sensitivity (Pud et al., 2005). Therefore, it is occasionally utilized as an experimental measurement of human endogenous pain suppression pathways in humans (Pud et al., 2009; van Wijk and Veldhuijzen, 2010). In brief, by comparing the pain sensitivity in different parts of the body, CPM measures the conditioned pain conditioning during or after painful stimuli (i.e., conditioned stimuli and test stimulus) and in the absence of conditioned stimuli (Yarnitsky et al., 2010). Opioid decreasing the inhibition of pathway activation has been suggested as an underlying mechanism for CPM, as opioid-treated subjects experienced reduced CPM (Ram et al., 2008). Therefore, some studies suggested that CPM and EIH may have a common mechanism. For example, in populations with and without chronic pain, a considerable correlation was found between the magnitude of CPM and EIH, indicating that CPM and EIH may activate the same downward inhibitory pathway (Fingleton et al., 2017; Lemley et al., 2015). Meanwhile, studies advocating the opposing viewpoint contended that the time course and spatial distribution of CPM and EIH are different (Rice et al., 2019). When a rise in pain threshold was observed only during and immediately after conditioned stimulation, EIH lasted longer (about 15 min) than CPM (Vaegter et al., 2014). In addition to differences in time course, their analgesic effects present a different spatial distribution. For example, CPM has greater analgesia effects in non-localized sites than in localized sites. Conversely, EIH has a greater effect of analgesia in local sites than in non-localized sites (Vaegter et al., 2014). In conclusion, the research highlighted the possibility that the mechanisms underlying conditioned pain regulation and EIH are distinct.

The discomfort in BFRT is similar to that in HL-RT (Martín-Hernández et al., 2017; Mattocks et al., 2019). The increase in discomfort during BFRT may act as a conditioned stimulus and trigger CPM (Hughes and Patterson, 2019). In comparison to LL-RT and HL-RT without BFRT, BFRT caused an increase in PPTs and muscle discomfort (Hughes and Patterson, 2020). In a recent study, there was a significant association between muscle discomfort and pain. This suggesting that CPM may be triggered after BFRT. However, the PPT in this study remained higher than baseline 24 h after exercise under BFRT, which could not be explained by CPM because muscle soreness due to exercise is unlikely to affect outcomes 24 h after exercise (Hughes and Patterson, 2020). This result led to the conclusion that local mechanisms, as opposed to CPM, are primarily responsible for prolonged hypoalgesia. However, CPM remains one of the underlying mechanisms of BFRT in the treatment of knee pain.

## 4 Conclusion and future directions

**FIGURE 1:**
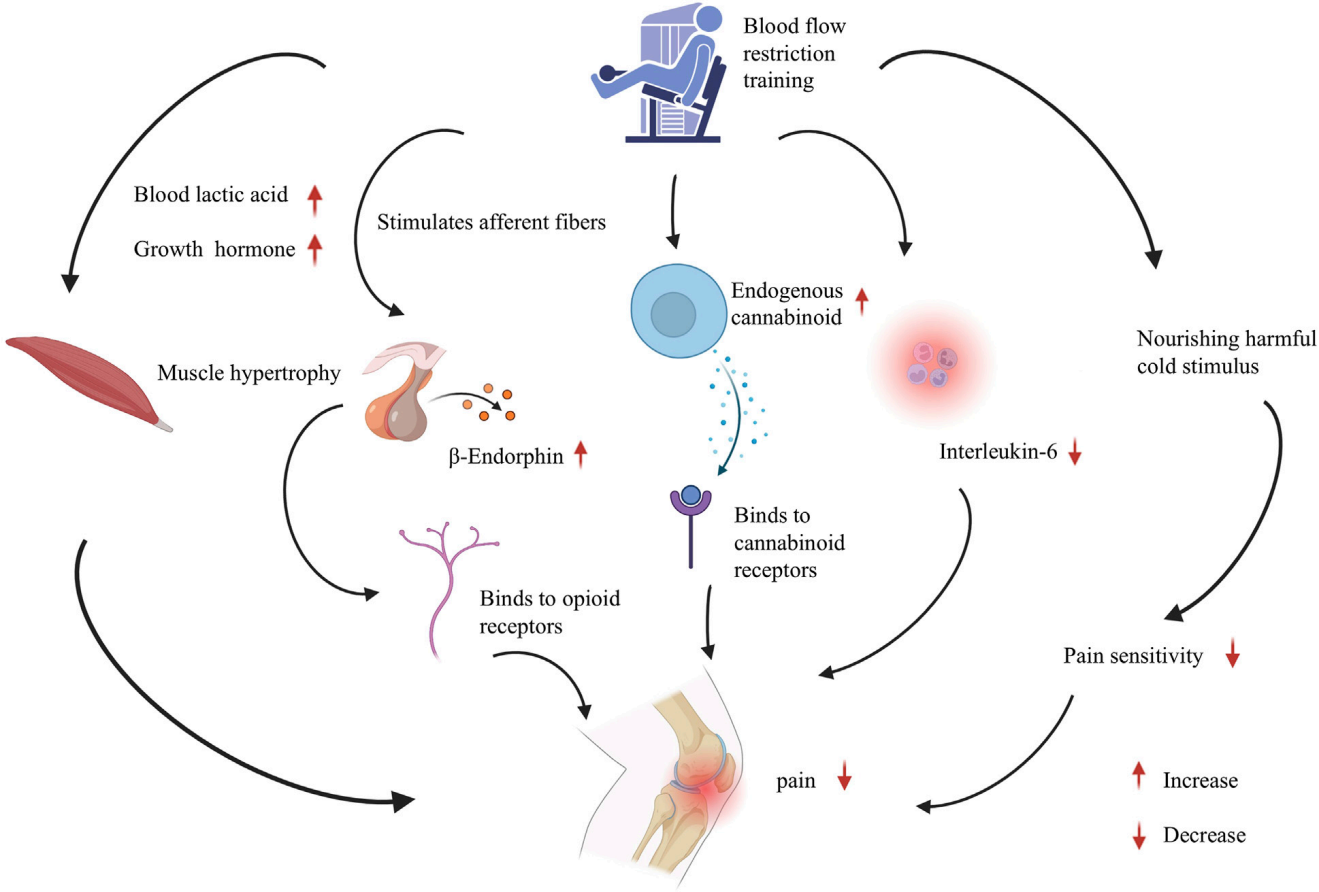
The involved mechanisms in blood flow restriction training on knee pain. The analgesic mechanism of blood flow restriction training for knee pain, involving muscle hypertrophy, endogenous opioid system, endocannabinoids, inflammation regulation, and conditional pain regulation. Abbreviations: BFRT, Blood flow restriction training; BE, Beta-endorphin; ECB, Endogenous cannabinoid; IL-6, nterleukin-6.

This review delves into the underlying mechanisms of BFRT (Figure 1). BFRT may hold promise for patients with KOA and ACL injuries; however, additional research is warranted, particularly high-quality, large-sample randomized controlled trials. A thorough understanding of the mechanism of BFRT is essential for developing personalized rehabilitation programs tailored to patients with various conditions.

At present, only one study has examined the changes of BE and 2-AG in patients after BFRT, without valuable findings. The limited studies could not fully explain that EIH and ECB are the potential mechanisms of BFRT in the treatment of knee pain. The relationship between another endogenous ligand agonist (anandamide) and BFRT has yet to be studied to fill the gap between BFRT and ECB. A substantial body of high-quality randomized controlled trials is warranted in future research to investigate underexplored areas, particularly focusing on anandamide levels and CPM mechanisms. Additionally, further studies should examine the long-term outcomes of BFRT and its effects across various age groups.
